# Biofabrication of Silver Nanoparticles (AgNPs) Using Embelin for Effective Therapeutic Management of Lung Cancer

**DOI:** 10.3389/fnut.2022.960674

**Published:** 2022-08-04

**Authors:** Rutika R. Jagtap, Aniket Garud, Shubhangi S. Puranik, Mithun Rudrapal, Mohammad Azam Ansari, Mohammad N. Alomary, Meshal Alshamrani, Ahmad Salawi, Yosif Almoshari, Johra Khan, Bhagyashri Warude

**Affiliations:** ^1^Post Graduate Research Centre, Department of Zoology, Modern College of Arts, Science and Commerce, Pune, India; ^2^Rasiklal M. Dhariwal Institute of Pharmaceutical Education and Research, Pune, India; ^3^Department of Epidemic Disease Research, Institute for Research and Medical Consultations (IRMC), Imam Abdulrahman Bin Faisal University, Dammam, Saudi Arabia; ^4^National Centre for Biotechnology, King Abdulaziz City for Science and Technology (KACST), Riyadh, Saudi Arabia; ^5^Department of Pharmaceutics, College of Pharmacy, Jazan University, Jazan, Saudi Arabia; ^6^Department of Medical Laboratory Sciences, College of Applied Medical Sciences, Majmaah University, Al Majma'ah, Saudi Arabia; ^7^Health and Basic Sciences Research Center, Majmaah University, Al Majma'ah, Saudi Arabia

**Keywords:** biofabrication, silver nanoparticles, embelin, anticancer, lung cancer, MTT assay, apoptosis assay

## Abstract

Nanobiotechnology is a burgeoning field of research with applications in cancer treatment, targeted chemotherapy, and molecular diagnosis. This study aims at the fabrication of silver nanoparticles using embelin derived from *Embelia ribes* to evaluate its anticancer property. Silver nanoparticles (AgNPs) have emerged as a novel nano-carrier for therapeutic agents with a wide range of medical capabilities due to their unique structural, physicochemical, and optical features. In our study, the particle size of fabricated AgNPs was measured as 25 nm, and the zeta potential was recorded as −5.42 mV, which indicates the good stability of embelin-derived AgNPs. The crystalline surface morphology was observed by SEM analysis. The FT-IR spectrum confirmed the reduction in silver ions (Ag^+^) by embelin, and the TEM analysis exhibited polydispersed Ag^+^ of 20–30 nm. The anticancer potential of embelin-fabricated AgNPs was investigated using *in vitro* studies on lung cancer cells by the MTT assay. The results revealed significant dose-dependent inhibition of cell proliferation against A549 cell lines. Embelin AgNP-induced apoptosis was measured by the annexin-V PI apoptosis assay, which exhibited significantly low necrotic cells as compared to apoptotic cells. Finally, the findings of our study suggest the anticancer potential of biofabricated embelin AgNPs, particularly against lung cancer cells.

## Introduction

The development of nanotechnology, which offers remarkable solutions to cope with life-threatening disorders, has boosted advancement in the field of medical science (1). Nanotechnology is a significant milestone that has numerous applications in a variety of fields, including electronics (2), textiles (3), cosmetics, and, most crucially, healthcare as targeted drug delivery, diagnosis, treatment, and biosensing for the benefit of humanity (4). Nanoparticles are an appealing platform for a wide range of biological applications. They are more precise therapeutic strategies for difficult-to-control disorders such as cancer.

There are a variety of metal-based nanoparticles, including gold, silver, zinc, iron, titanium, and magnesium (5). Among them, silver nanoparticles are fascinating due to their biomedical applications; silver can upregulate or downregulate cellular mechanisms as well as act as a medium to detect and diagnose body problems (6), and silver has been used since ancient times in ayurvedic medicines. Silver nanoparticles are used in wound dressing due to antimicrobial nature as well as an anticancer agent (7). The synthesis of nanoparticles is a major aspect as various methods are available, *viz*., chemical, physical, and biological. The alternative to the harmful chemical and physical method for the synthesis of the nanoparticle is “green synthesis”; an eco-friendly and cost-effective approach. Biological systems, such as plants and microorganisms, operate as reducing and capping agents in green synthesis, converting metal ions into metal nanoparticles without the use of complex instruments or chemicals (8). Plants, being a significant reservoir of phytoconstituents, act as a reducing, stabilizing, and capping agent in the creation of nanoparticles.

Cancer is a diverse category of diseases that are fatal and characterized by uncontrolled cellular development. In a multistage linear process, cancer cells progress from a precancerous lesion to a heterogeneous malignant tumor capable of spreading to other organs (9). According to the World Health Organization, cancer is the world's top cause of death, accounting for ~10 million deaths in 2020 (10). Lung cancer is one of the leading causes of death among all cancers; it accounted for ~1.8 million deaths in 2020 (10). Based on clinicopathological stages and cytology, lung cancer can be divided into small cell lung carcinoma (SCLC) and non-small cell lung carcinoma (NSCLC) (11, 12). The rise of lung cancer cases is due to tobacco/cigarette smoking, and it is the single most factor accounting for up to 90% of all lung cancer cases. Other factors responsible for causing lung cancer are radon, a radioactive gas released during the decay of uranium, thorium, and radium; asbestos, the heat and corrosion resistive fibrous material; environmental tobacco smoke and air pollutants, such as arsenic, nickel, and benzo[a]pyrene (13). A hereditary predisposition to lung cancer has been linked to a 1.7-fold increased chance of developing lung cancer. Infecting agents such as human papillomavirus (HPV) and Epstein-Barr virus are also linked with lung cancer (13, 14).

Despite substantial advances in disease biology and many therapeutic tools such as surgery, radiation therapy, chemotherapy, and targeted therapy, successful cancer care remains elusive (15). Conventional therapies are unsuitable because of a large number of side effects, nonspecificity, high treatment costs, recurrence, and cancer spread (16). As a result, there is a strong need to find a therapeutic treatment that is distinctive, target-oriented, safe, and low-cost. The advancement of medical science has been accelerated by the development of nanotechnology, which offers extraordinary methods for dealing with life-threatening illnesses.

Medicinal plants have been used to treat diseases since antiquity, and they constitute an essential component of traditional healthcare systems throughout the world. The creation of medicinal products and medications has remained a major source of hope for treating a number of human degenerative conditions, such as cancer (17). A large range of medicinal plants has been tested for their broad-spectrum anticancer properties. Eventually, anticancer medicines such as paclitaxel, vincristine, vinblastine, vinorelbine, and camptothecin were isolated as a result of this effort (18). The major research goal in the field of medicinal plants is to address the nature of medicinal plants in cancer therapy in a comprehensive and integrative approach.

**Figure 1:**
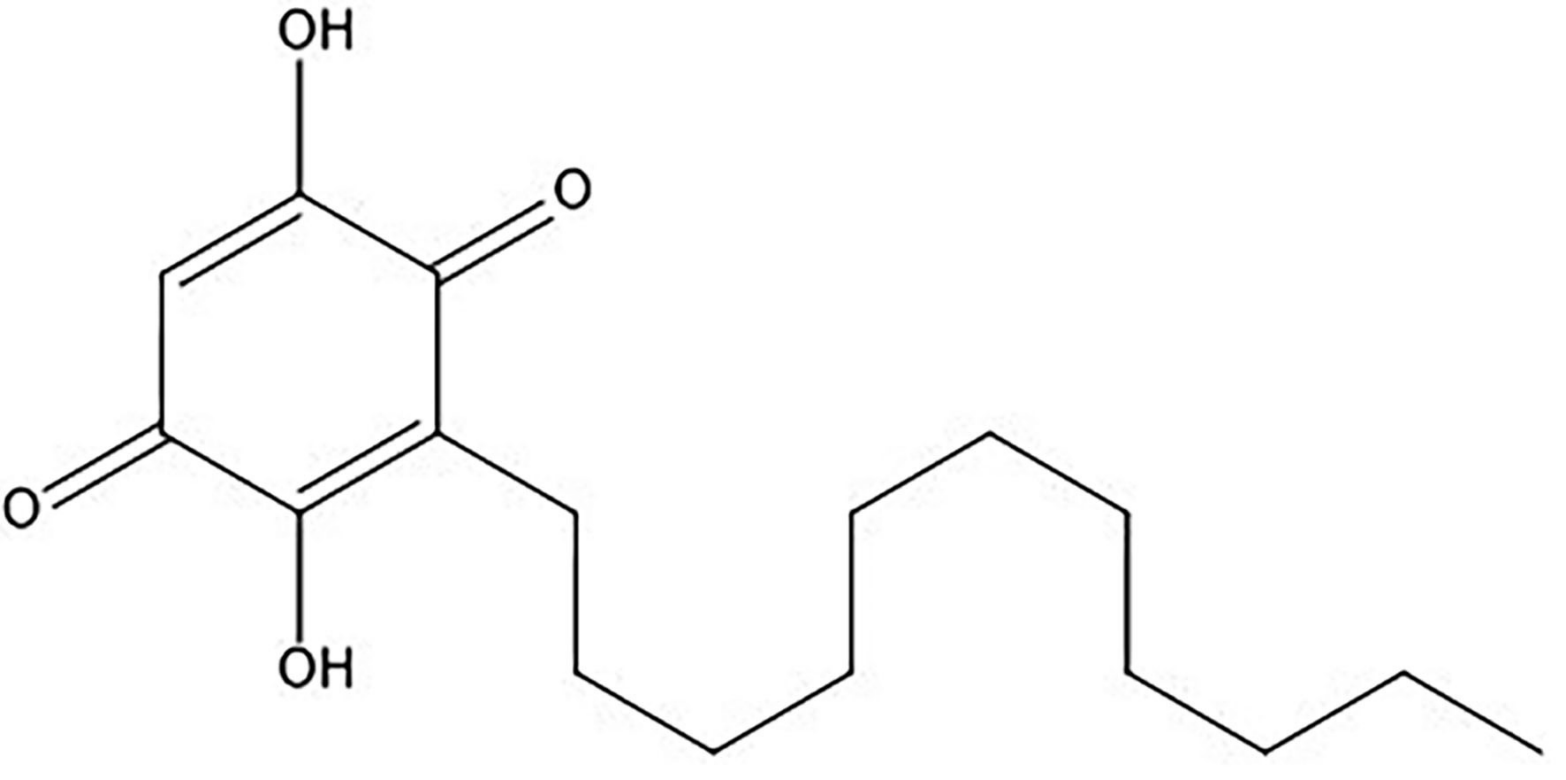
Chemical structure of embelin.

*Embelia ribes* is a traditional medicinal plant that belongs to the Myrsinaceae family and is frequently used in Ayurvedic medicines. It is most recognized for its antihelmentic activity, which is called *Krmighna*. It has anti-inflammatory, antioxidant, cytotoxic, antimicrobial, antifungal, and wound-healing properties (19). Embelin (2,5-dihydroxy-3-undecyl-1,4-benzoquinone, molecular weight: 294.4) (Figure 1) is a strong quinine-derivative phytoconstituent of *E. ribes* (especially in fruits) that has been extensively explored for its antihelmintic, antitumor (20), anti-inflammatory, antidiabetic, anticancer, and anticonvulsant properties (21). Embelin has anticancer benefits in the pancreas (22), colon (23), and liver tumors in animals, according to *in vivo* research (24–26). Thus, embelin is a potent molecule to consider for cancer treatment.

Silver metal nanoparticles, which exhibit amazing broad-spectrum activities, are the most extensively studied. Plants can synthesize silver nanoparticles; embelin is a phytoconstituent that can reduce the number of silver ions (27–29). In view of the preceding, we aimed to conduct the current work on embelin-aided production of silver nanoparticles, their characterization, and anticancer potential against A549 lung cancer cells. An annexin PI FITC assay was also carried out to investigate the effect of the embelin-derived silver nanoparticles in inducing apoptosis.

## Materials and Methods

### Isolation and Characterization of Embelin

*Embelia ribes* was collected from the Koyna region, Maharashtra, and embelin, a major phytoconstituent, was isolated from the fruits. Briefly, the fruits of *E. ribes* were dried, coarsely pulverized, and extracted in chloroform using Soxhlet apparatus. By eluting the extract with benzene, embelin was isolated using silica column chromatography (30). The isolated embelin was characterized by a chemical test, FT-IR, and ^1^H NMR spectral analyses.

A chemical test was performed for confirmation of embelin. Embelin was dissolved in pet ether, and diluted ammonia was added to the solution. The presence of embelin was indicated by a bluish-violet precipitate (31).

The FT-IR spectrum was obtained using an FT-IR spectrophotometer (Bruker, Germany). The IR spectrum was recorded by scanning over a wavelength of 400–4,000 cm^−1^ using the Origin software. The characteristic IR peaks were observed and compared with the reference spectrum of embelin (31). ^1^H NMR was done by a sophisticated NMR spectrophotometer (Bruker), and the ^1^H frequency was 500 MHz.

### Synthesis of Silver Nanoparticles (AgNPs)

A solution of 1 mM aqueous silver nitrate (AgNO_3_) was added to the embelin solution and vigorously stirred, and stirring was continued for 30 min to get colloids. The tubes were kept in the dark for the reaction to proceed further. The color of the AgNO_3_ solution changes from colorless to light brown after 30 min when embelin is added to it. After 24 h of incubation at room temperature, the hue deepens and turns to a dark reddish-brown tone (32). The AgNPs were synthesized by a process of reduction aided by embelin.

### Characterization of AgNPs

Different factors influence the features of synthesized nanoparticles, and there are a variety of characterization techniques for studying the traits and properties of nanoparticles.

#### UV-Visible Spectroscopy

As nanoparticles have unique optical properties, UV-visible spectroscopy is used to characterize AgNPs. The UV-visible spectrum was analyzed in the range of 200–800 nm in the UV-visible spectrophotometer (Shimadzu) 30 min after the addition of embelin solution with vigorous stirring (32).

#### Particle Size and Zeta Potential Analysis

The particle size of synthesized nanoparticles was analyzed by using the NANOPHOX-SympaTec (Germany) apparatus. The required analysis volume ranges between 50 μl and 4 ml, and the size range of scattering is 0.5–10,000 nm. The zeta potential of synthesized AgNPs is analyzed using Delsa Nano C by Beckmen Counter Inc. in order to analyze the stability of NPs. The liquid sample was diluted 10 times with distilled water and centrifuged. The zeta potential of the generated AgNPs was assessed in the presence of water as dispersion medium (33).

#### Fourier Transform Infrared (FT-IR) Spectroscopic Analysis

The existence of numerous reducing and stabilizing functional groups of embelin was confirmed using an infrared spectrum, as well as their likely role in the manufacture of AgNPs. FT-IR (Bruker) was used to investigate the functional group responsible for the AgNPs in the wavelength range from 4,000 to 400 cm^−1^ (32).

#### Transmission Electron Microscopy (TEM)

The morphology, size, and shape of embelin biofabricated AgNPs were determined by TEM analysis. The dispersed solution of AgNPs was sonicated for 20 min, and the TEM grid was prepared by placing a drop of diluted solution on a carbon-coated grid and later drying overnight. TEM measurements were done by a JEOL JEM-2100 (JEOL, Peabody, MA, USA) high-resolution transmission electron microscope (33).

#### Field Emission Scanning Electron Microscopy (FESEM)

A FESEM is used to visualize extremely fine topographic characteristics on the surface of whole or fractioned objects. The morphology and shape of the AgNPs were examined using field emission electron microscopy (Icon Analytical, Quanta 250, FEI, United States). The AgNPs suspension was air-dried, loaded to the sample holder, and coated, and images were obtained at 20 kV with a different magnification (32).

### Evaluation of Anticancer Activity

#### Cell Viability Study by MTT Assay

Human lung cancer cell line A549 was procured from the National Centre for Cell Science (NCCS), Pune (India). The cells were maintained in Dulbecco's modified Eagle medium (DMEM) supplemented with 10% fetal bovine serum (Gibco1X) and antibiotic Antimycotic (Gibco). The cell line was maintained at 37 C in a humidified atmosphere of 5% CO_2._

The 3-(4,5-dimethylthiazol-2-yl)-2,5-diphenyltetrazolium bromide (MTT) assay was used to assess cell viability. Adhered cells were trypsanized and seeded in 96-well plates after detaching from the surface. After allowing the cells to adhere for 24 h, they were treated in triplicate with the relevant drug concentrations for another 24 h. Each well was filled with 0.5 mg/ml MTT and covered with aluminum foil. For 4 h, plates were incubated at 37°C. Following the incubation period, the culture medium was withdrawn from each well and dimethyl sulfoxide (DMSO) was applied to dissolve the blue-purple formazan crystals. At 570 nm, the absorbance of the blank, control, and treatment wells was measured on a microplate reader (26).

### Annexin PI Apoptosis Assay

The FITC annexin V/dead cell apoptosis kit (catalog no. V13241, Invitrogen) containing FITC annexin V and propidium iodide (PI) was used to assess embelin AgNPs-mediated apoptotic induction, as directed by the manufacturer (34). Briefly, cells were cultured and treated with drugs and incubated. Phosphate-buffered saline (PBS) was used to wash the cells after they were triggered to apoptosis. The cells were then resuspended in an annexin-binding buffer. The cells were stained with FITC annexin V and PI and incubated at room temperature for 15 min and then washed. After the incubation period, cells were resuspended in annexin-binding buffer and kept on ice until analyzed by a flow cytometer (Attune NxT acoustic flow cytometer Invitrogen, using the Attune nxt software v. 2.1) (35). Flow cytometry was used to examine both untreated (negative control) and positive control cells (doxorubicin with 6 g/ml-treated cells).

## Results and Discussion

### Isolation and Characterization of Embelin

Embelin was isolated from *E. ribes* berries/fruits by the hot percolation method. Chloroform was a solvent required to isolate the embelin through Soxhlation. Embelin is a benzoquinone, and the presence of a long alkyl chain makes it a nonpolar compound. Hexane as an extraction solvent has been reported earlier, but it shows impurities of other phytochemicals during purification. The purification of embelin was done through column chromatography using a silica column, and on elution with benzene, embelin flakes were recovered. Silica is more polar than the mobile phase benzene and hence embelin being nonpolar is carried by benzene more readily during elution (30).

The purity of embelin was confirmed by physical appearance such as color, consistency, and chemical tests. The color of embelin was observed as golden orange, and flakes were crystalline. The chemical test was performed to indicate the presence of embelin; the formation of a bluish-violet precipitate confirmed the presence of embelin. Further characterization was done by FT-IR and ^1^H NMR analyses.

The FT-IR analysis revealed major functional groups of embelin and was analyzed using the Origin Pro software and is shown in Supplementary Figure 1. The observed peaks in the IR spectrum of isolated embelin were in accordance with the functional groups of standard embelin (31).

Proton nuclear magnetic resonance is used for the structural determination of molecules. The proton NMR (^1^ H NMR−500 MHz, CDCl_3_) spectrum revealed chemical shifts at δ 7.65 (2H, broad spectra, 2× OH), 6.0 (1H, singlet), 2.43–2.46 (2H, triplet), 1.46–1.47 (2H, triplet), 1.26–1.30 (4H, broad spectra), 1.26 (12H, broad spectra), and 0.86–0.89 (3H, triplet). ^1^H NMR of isolated embelin is shown in Supplementary Figure 2.

### Synthesis and Characterization of AgNPs

#### Synthesis of AgNPs

The colorless AgNO_3_ solution turned pinkish brown and colloids developed after the addition of embelin to AgNO_3_. The color intensified to brown after 24 h of incubation at room temperature (32). The reduction of silver nitrate to elemental silver (Ag^+^ to Ag^0^) was due to the interaction of AgNO_3_ with embelin.

#### Characterization of AgNPs

##### UV-Visible Spectroscopy

The UV/vis spectrum of AgNPs synthesized in the range of 200–800 nm gave the maximum absorption peak at 384.5 nm. The conjugated system of embelin affects its UV/vis spectral profile. The presence of free electrons in metal nanoparticles produces a surface plasmon resonance (SPR) absorption band due to collective oscillation in resonance with the light wave (32). The synthesized AgNPs were analyzed using a UV-visible spectrophotometer (Shimadzu). The absorption spectrum is shown in Supplementary Figure 3.

##### Particle Size and Zeta Potential

For the determination of the surface charge and stability of the formulation, a zeta potential analysis is carried out (33). Measuring the velocity of the nano-sized particles also assesses the colloidal stability of AgNPs. A distinct peak between 25 and 30 nm clearly indicates the formation of AgNPs with uniformity. The higher peak value indicates the monodispersity of nanoparticles. The zeta potential of −5.42 mV was recorded, which indicates the good stability of AgNPs (Supplementary Figures 4A,B).

##### Fourier Transform Infrared Spectroscopy

The FT-IR analysis of AgNPs is used to identify the molecules that act as coating and stabilizing agents, as well as to detect silver ion reduction (31, 32). The FT-IR spectrum of AgNPs (Supplementary Figure 5) synthesized using embelin showed sharp absorption peaks at 952.84, 1,029, and 1,043 cm^−1^ corresponding to secondary alcohol ring stretching. A broad peak in between 3,340 and 3,588 cm^−1^ is characteristic of the presence of the hydroxyl group (OH) of biomolecules present in the synthesized AgNPs. The various peaks represent biomolecules attached to AgNPs and reduce Ag^+^ to AgNPs by acting as capping and stabilizing agents.

##### Field Emission Scanning Electron Microscopy

**Figure 2:**
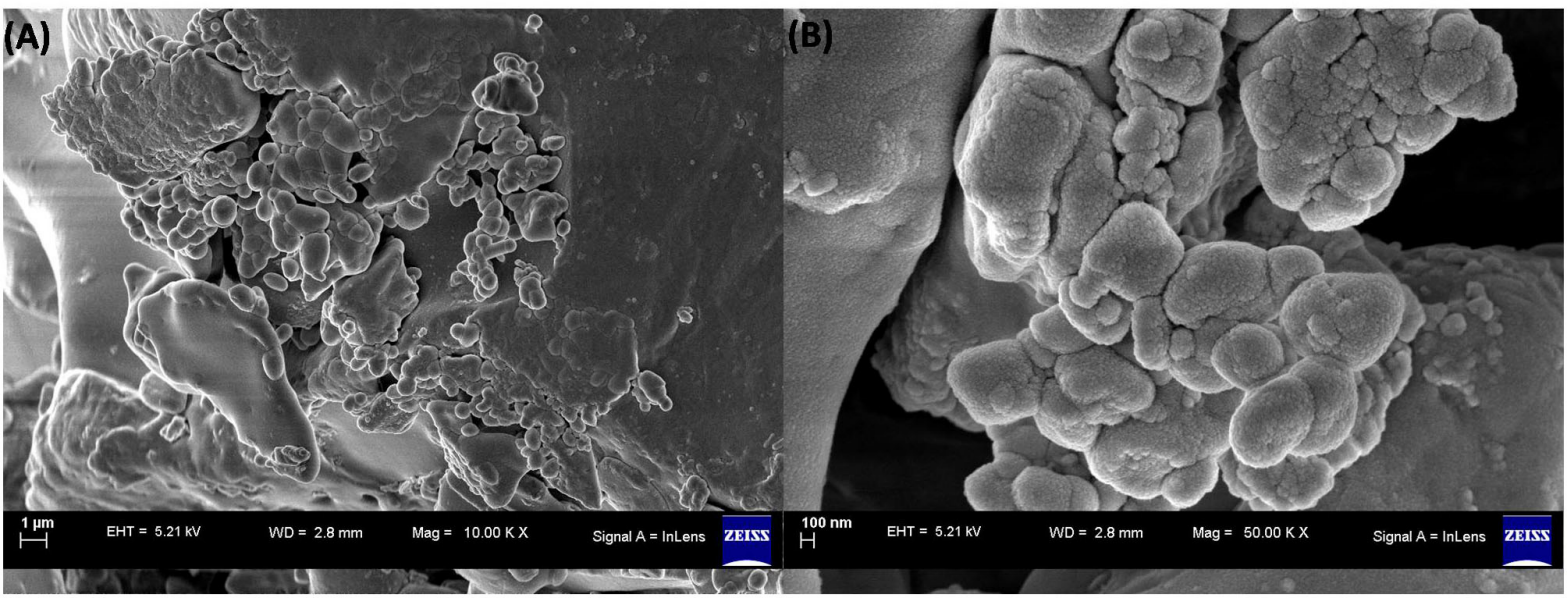
FESEM images of embelin-derived AgNP. **(A)** 1 μm and **(B)** 100 nm.

The image of a nanoparticle's surface at high resolution provides useful information such as size, shape, topography, composition, electrical conductivity, and other properties. The FESEM images of the embelin-derived silver nanoparticles are shown in Figures 2A,B. The surface morphology of AgNPs showed an even shape and spherical nature. The particle size ranges from 20 to 30 nm.

##### Transmission Electron Microscopy

**Figure 3:**
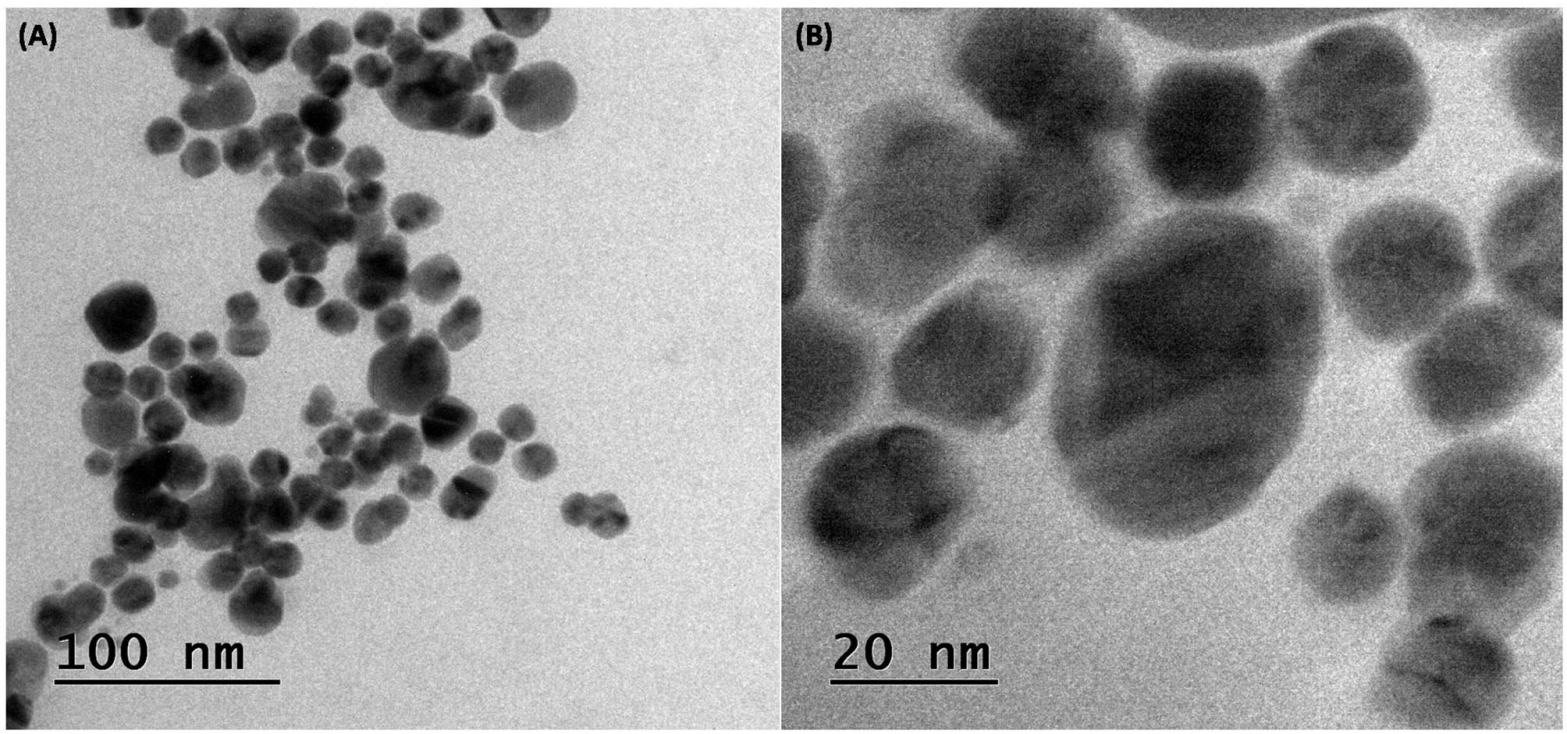
TEM images of embelin-derived AgNP. **(A)** 100 nm and **(B)** 20 nm.

The TEM provides structural and chemical behavior of nanoparticles under high electron beam conditions with great resolution (1). The shape and size of the resultant AgNPs were analyzed with the help of TEM (Figures 3A,B). The AgNP solution was placed on a carbon-coated copper grid and allowed to dry, and TEM images were recorded. The sizes of AgNPs were ~20–30 nm as per the TEM micrographs and were found to be spherical.

### Anticancer Activity

#### Cell Viability

**Figure 4:**
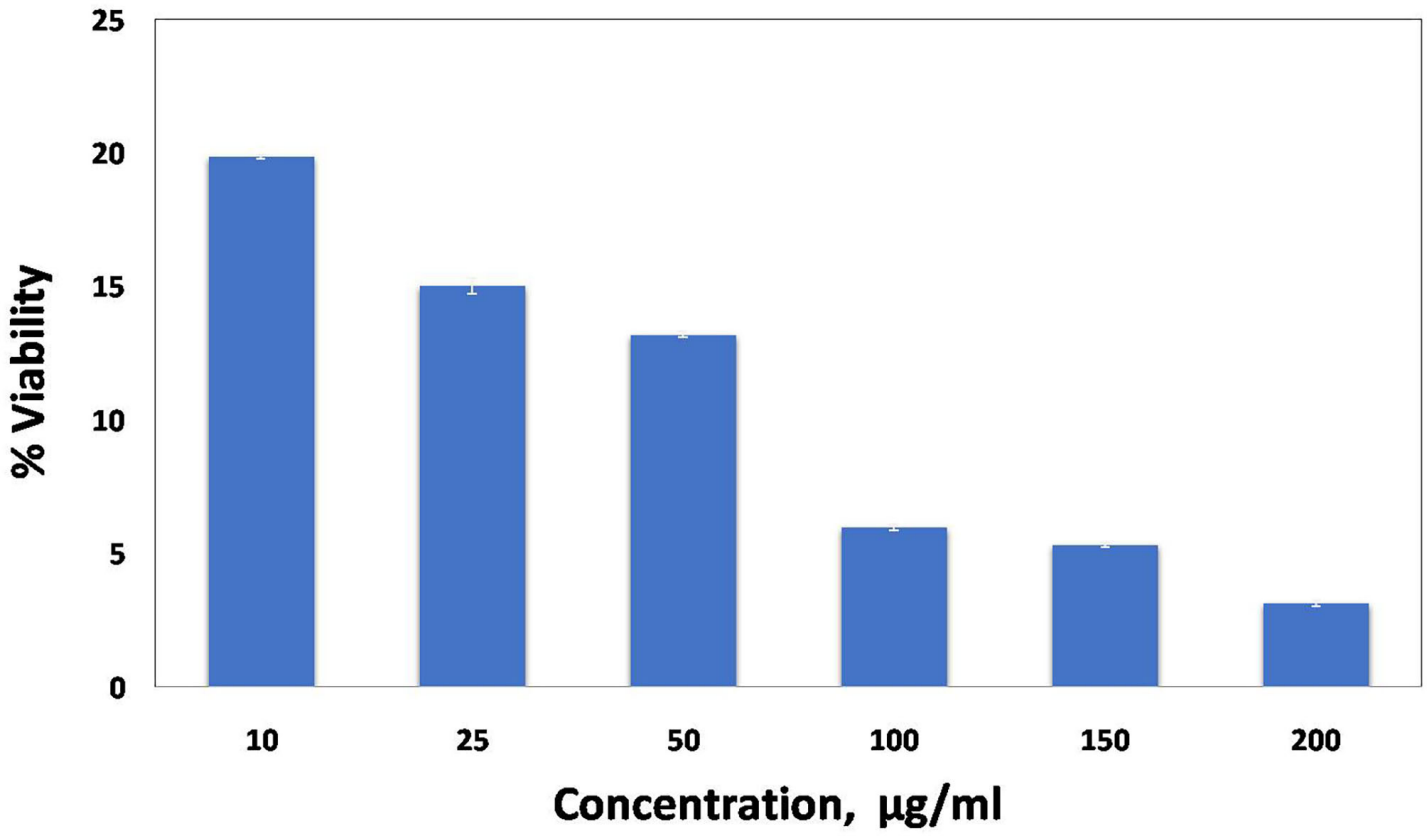
Cell viability by MTT assay. A549 cells treated with embelin AgNPs.

The cytotoxic potential of embelin-derived AgNPs against the human lung cancer cell line A549 was assessed by using the MTT assay, which is widely used in cytotoxicity and cell viability assays. For the cytotoxicity study, A549 lung cancer cells were incubated with different concentrations of embelin-aided AgNPs (10, 25, 50, 100, 150, and 200 μg/ml). After 24 h of incubation, cell viability was assessed by the MTT assay. It was observed that the effects of embelin-derived AgNPs were in a dose-dependent manner. Embelin-based AgNPs could exhibit cytotoxicity at low doses. At 10 μg/ml concentration, % inhibition was 80.131 ± 0.068, whereas in doxorubicin 10 μg/ml, it was 89.364 ± 0.080. Doxorubicin, a standard anticancer drug, exerts antimitotic activity by intercalating between DNA base pairs. When compared, embelin-AgNPs showed slightly lower activity than doxorubicin. At 200 μg/ml, embelin AgNPs showed a % inhibition of 96.864 ± 0.112, suggesting an increase in % inhibition in a dose-dependent manner (Figure 4).

However, the exact molecular mechanism of cytotoxicity needs to be explored. As per Maeda 2003, the possible mechanism of nanoparticles in drug targeting is enhanced permeability and retention [EPR]. The EPR results in the passive accumulation of medications and drug carriers due to extravasation through leaky vasculature. Nano-carriers or nanoparticles supposedly use the same EPR mechanism to deliver the anticancer drugs at the targeted site (35).

Embelin is a phytoconstituent and is therefore considered safer for normal cells, and at the same time, it exhibits cytotoxicity against cancer cells. Silver has been used since ancient times in Ayurveda, which possesses anticancer properties (1, 36). The combination of Ag^+^ and embelin acted upon A549 cells to show cytotoxicity. Mahendran et al. (38) synthesized AgNPs from embelin and studied their cytotoxicity against human MG-63 (osteosarcoma cells). In their study, they found that the AgNPs derived from embelin were efficient to deliver embelin to cancer cells and showed cytotoxicity (37, 38). The study also suggested that embelin AgNPs were more efficient than pure embelin due to the water solubility of embelin AgNPs and the presence of Ag^+^ ions.

#### Annexin PI Apoptosis

**Figure 5:**
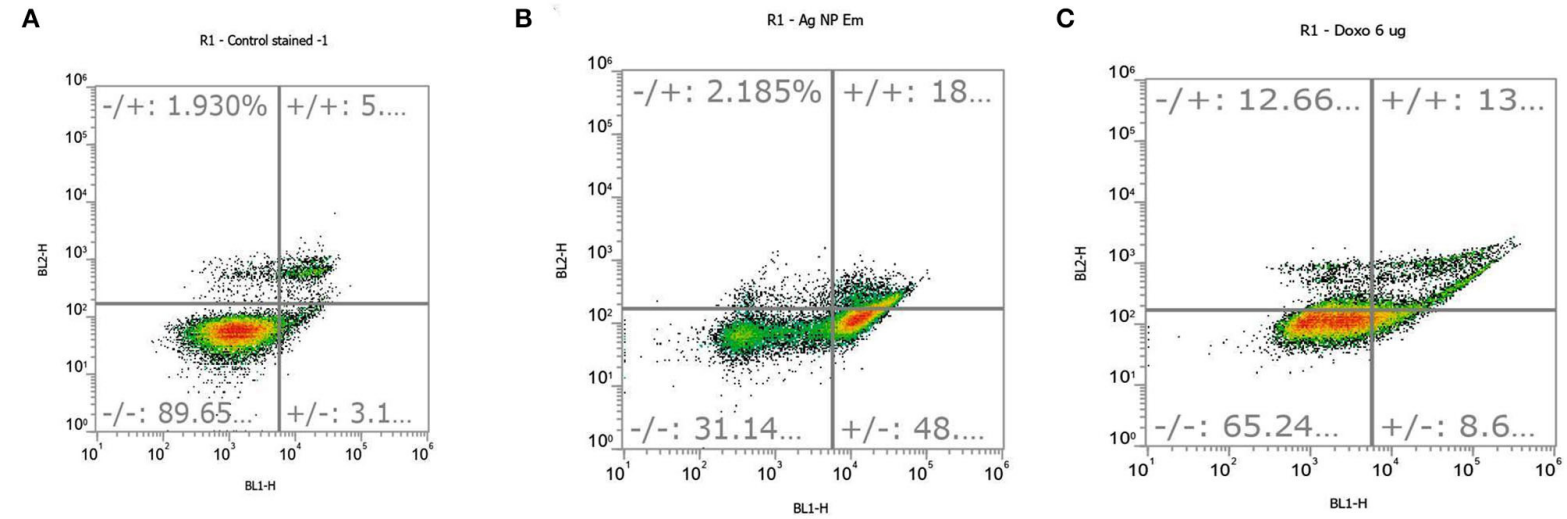
Apoptosis occurred in **(A)** control cells, **(B)** embelin AgNP-treated cells, and **(C)** doxorubicin (standard)-treated cells at a concentration of 6 μg/ml. Quadrants are as follows: Q1, necrotic cells; Q2, late apoptotic cells; Q3, viable cells; and Q4, early apoptotic cells.

For embelin-based AgNPs, the IC_50_ value was used in triplicate to treat human lung cancer A549 cells, and it exhibited cytotoxic action. Cells were stained with FITC-labeled annexin V and PI dyes after apoptosis induction, and the cell suspension was analyzed by flow cytometry. The annexin V assay revealed that embelin-derived AgNPs induced early and late stages of apoptosis in A549 cells. The annexin V-FITC graphs in Figure 5 show the distribution of A549 cells in four quadrants (Q1, Q2, Q3, and Q4), and it is one of three independent studies performed.

In comparison to control cells, all treated cells showed a lower percentage of viable cells. There was very less cell dispersion in Q1, Q2, and Q4 in the control cells, indicating a very low amount of necrotic, late, and early apoptotic cells, respectively. After treatment with embelin-based AgNPs, the distribution of cells in these quadrants (Q1, Q2, and Q4) increased. As per the triplicate study, 48% of the embelin-derived AgNPs-treated cells were in the early stages of apoptosis (Q4), whereas 18% were in the late stages of apoptosis (Q2) (Figure 5B). Q1 denoted necrotic cells, which had a minor increase in cell dispersion.

**Figure 6:**
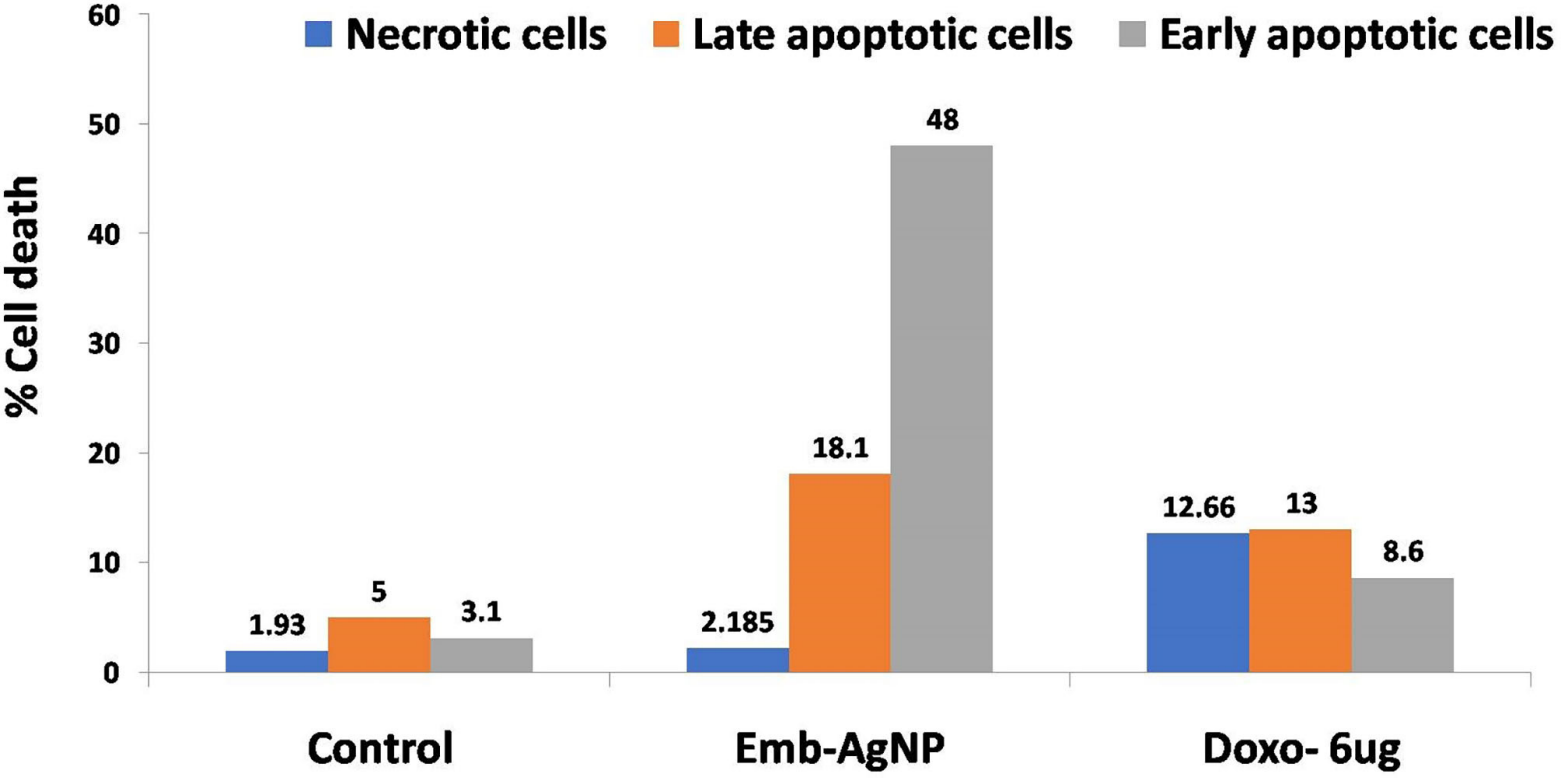
Annexin PI assay for apoptosis analysis. Emb, embelin; Doxo, doxorubicin.

In the control cells, only 1.93% of necrotic cells were observed along with 5% of late apoptotic cells (Figure 5A), while, in embelin AgNP-treated cells, necrotic cells were found to be 2.18%. In the positive control, cells were treated with doxorubicin, a well-known anticancer drug, at a concentration of 6 μg/ml, which resulted in increased apoptosis. Doxorubicin showed 13% of cells in early apoptosis, while 8.6% of cells in late apoptosis, but it also showed 12.66% of necrotic cells (Figure 5C). The graphical representation of the annexin PI apoptosis assay is shown in Figure 6. Embelin is a phytoconstituent; hence, embelin AgNPs resulted in less necrotic cells.

Apoptotic cell death is a closely regulated process characterized by morphological alterations in the cellular membrane structure. Cell shrinkage, blebbing of the plasma membrane, cell separation, phosphatidylserine translocation, nuclear condensation, and DNA fragmentation are all signs of apoptosis. Embelin AgNPs caused apoptosis in A549 cells, according to the current apoptotic investigation. Various underlying mechanisms can cause apoptosis to occur. The exact signaling pathway and the apoptotic process can be determined through a further investigation of the molecular mechanisms.

## Conclusion

The therapeutic potential of embelin of *E. ribes*-derived AgNPs in the treatment of lung cancer is the outcome of this investigation. The embelin fabricated AgNPs possess a substantial anticancer effect against A549 lung cancer cells. Further apoptosis assay reveals considerable induction of apoptosis in A549 cells by embelin AgNPs. The use of embelin in the formulation of AgNPs has opened new doors to design safer nanomedicine for the treatment of cancer with the application of nanotechnology. It is necessary to explore the possible cellular and molecular mechanisms along with toxicity issues of embelin AgNPs in future work. However, the amalgamation of the silver ions with embelin at the molecular nano level can work marvels and could be a viable therapeutic strategy in the management of cancer.

## Data Availability Statement

The original contributions presented in the study are included in the article/Supplementary Material, further inquiries can be directed to the corresponding author/s.

## Author Contributions

RJ, AG, SP, MR, and BW: conceptualization, methodology, software, investigation, writing-original draft, review and editing, resources, and supervision. MAn, MAlo, and MAls: validation and formal analysis. MAn, MAlo, MAls, AS, YA, and JK: funding acquisition. AS, YA, and JK: visualization and software. MR: critical analysis and final draft-review and editing. All authors contributed to the article and approved the submitted version.

## Conflict of Interest

The authors declare that the research was conducted in the absence of any commercial or financial relationships that could be construed as a potential conflict of interest.

## Publisher's Note

All claims expressed in this article are solely those of the authors and do not necessarily represent those of their affiliated organizations, or those of the publisher, the editors and the reviewers. Any product that may be evaluated in this article, or claim that may be made by its manufacturer, is not guaranteed or endorsed by the publisher.

## Supplementary Material

Supplementary Figure 1 FT-IR spectrum of embelin.

Supplementary Figure 2 ^1^H NMR spectrum of isolated embelin.

Supplementary Figure 3 UV-visible spectrum of embelin-derived AgNPs.

Supplementary Figure 4 **(A)** Cross-correlation for particle size analysis [NANOPHOX (NX0088)]; **(B)** zeta potential of embelin AgNPs.

Supplementary Figure 5 FT-IR spectrum of embelin-derived AgNPs.
