# Supplementary figures and images for: Biofabrication of Silver Nanoparticles (AgNPs) Using Embelin for Effective Therapeutic Management of Lung Cancer
Source: Front Nutr. 2022 Aug 4;9:960674. doi: 10.3389/fnut.2022.960674 (PMC9386231; doi:10.3389/fnut.2022.960674)

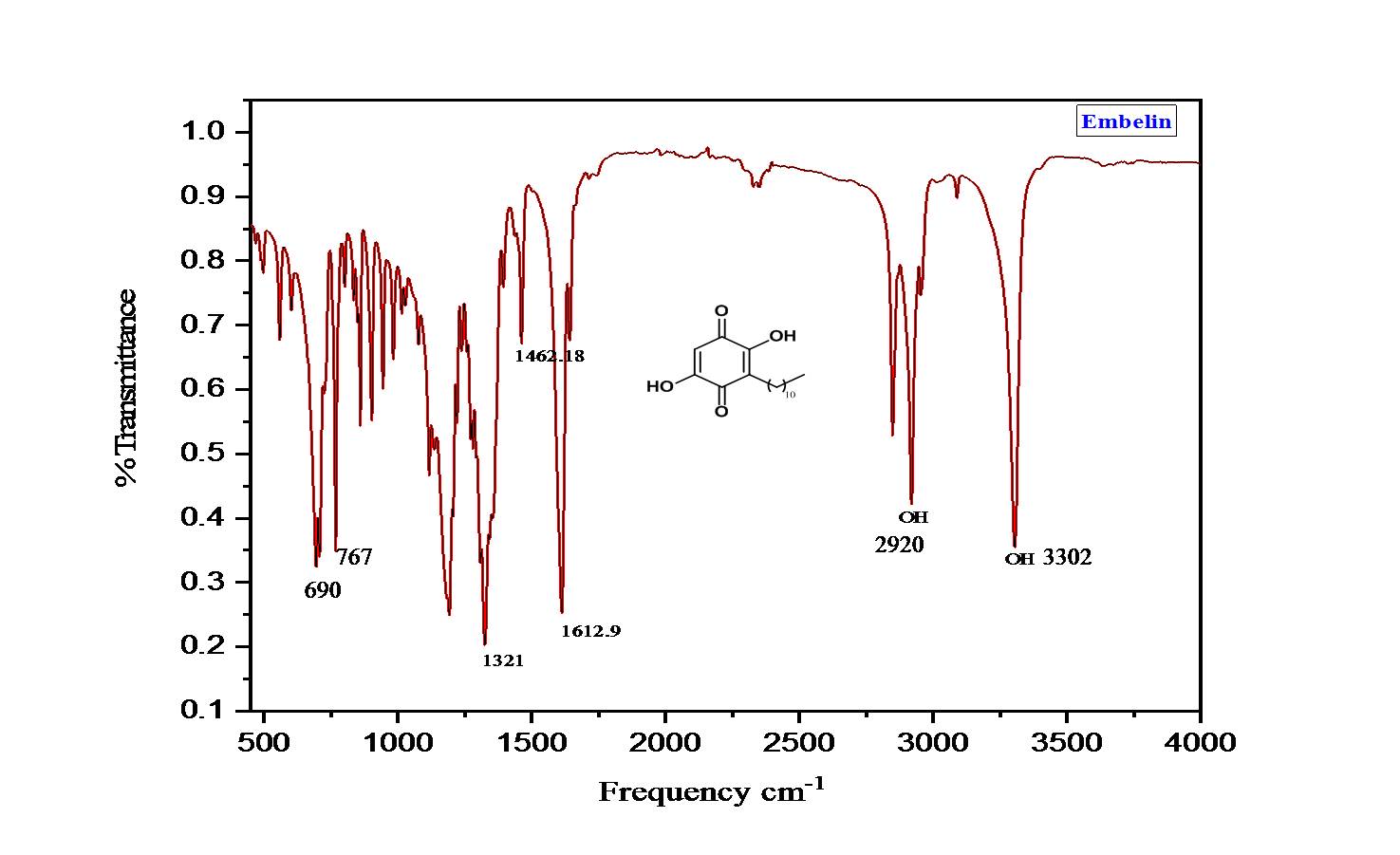

Supplement: Supplementary Figure 1 — FT-IR spectrum of embelin. [file Image_1.JPEG]

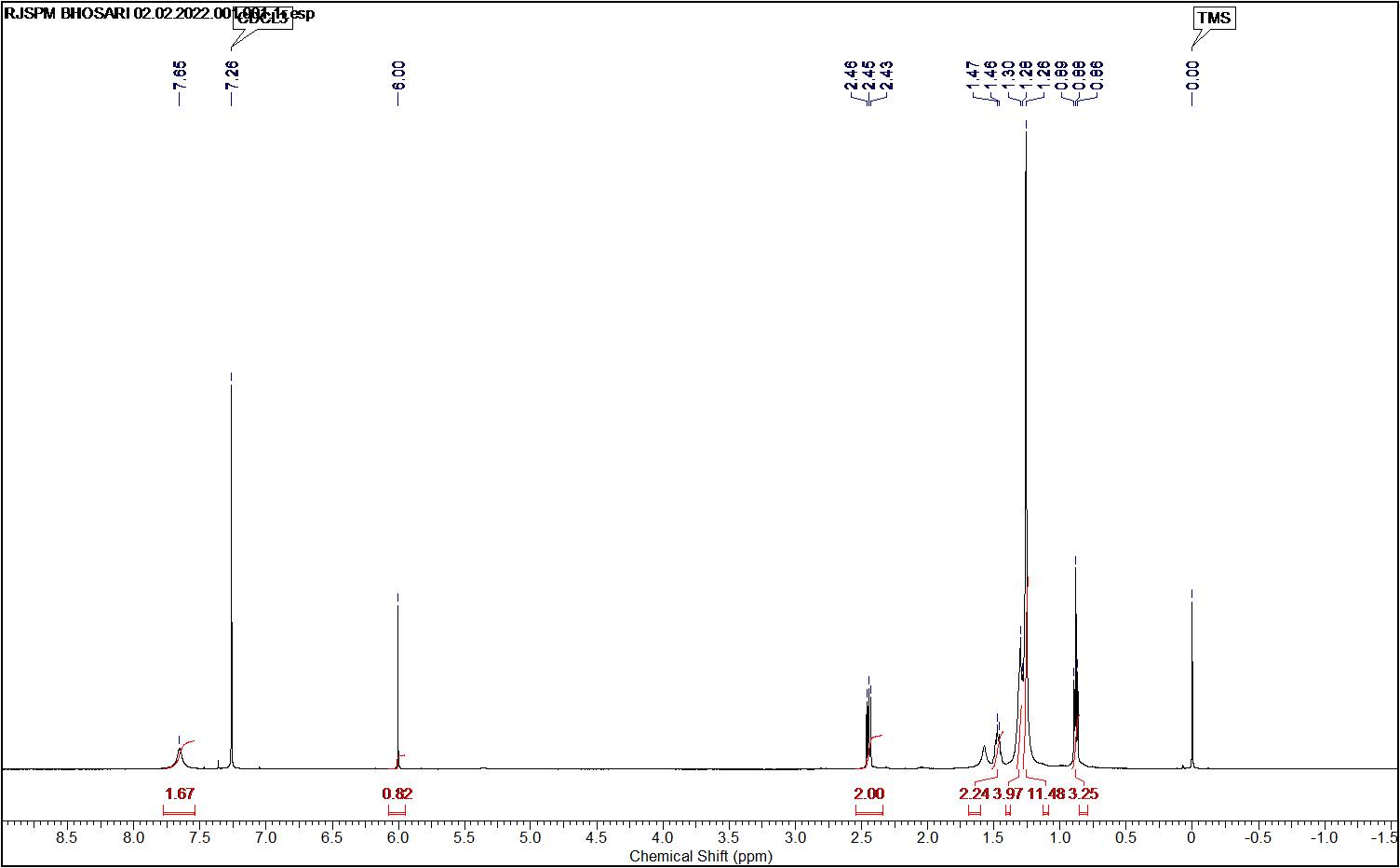

Supplement: Supplementary Figure 2 — 1H NMR spectrum of isolated embelin. [file Image_2.JPEG]

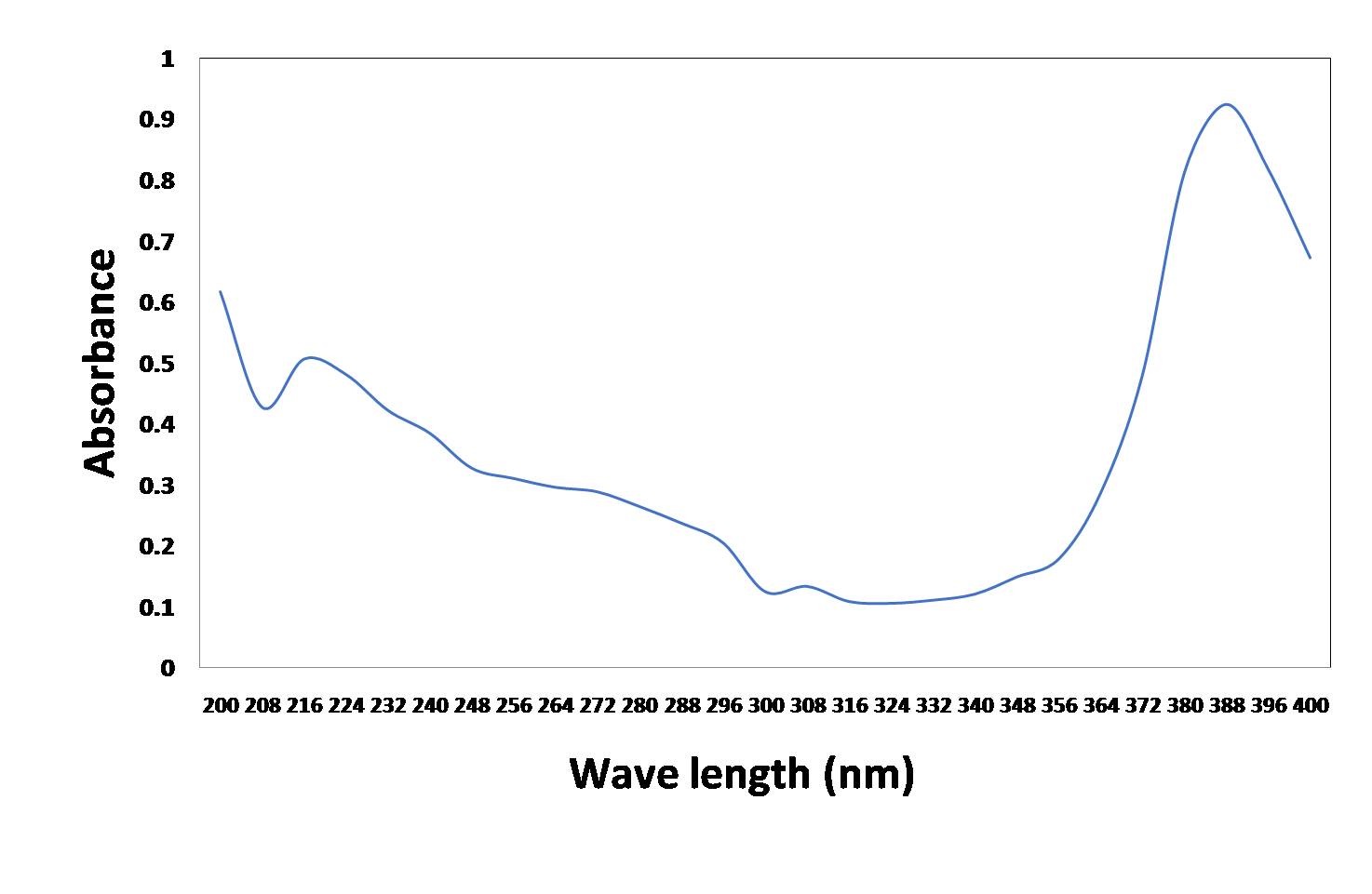

Supplement: Supplementary Figure 3 — UV-visible spectrum of embelin-derived AgNPs. [file Image_3.JPEG]

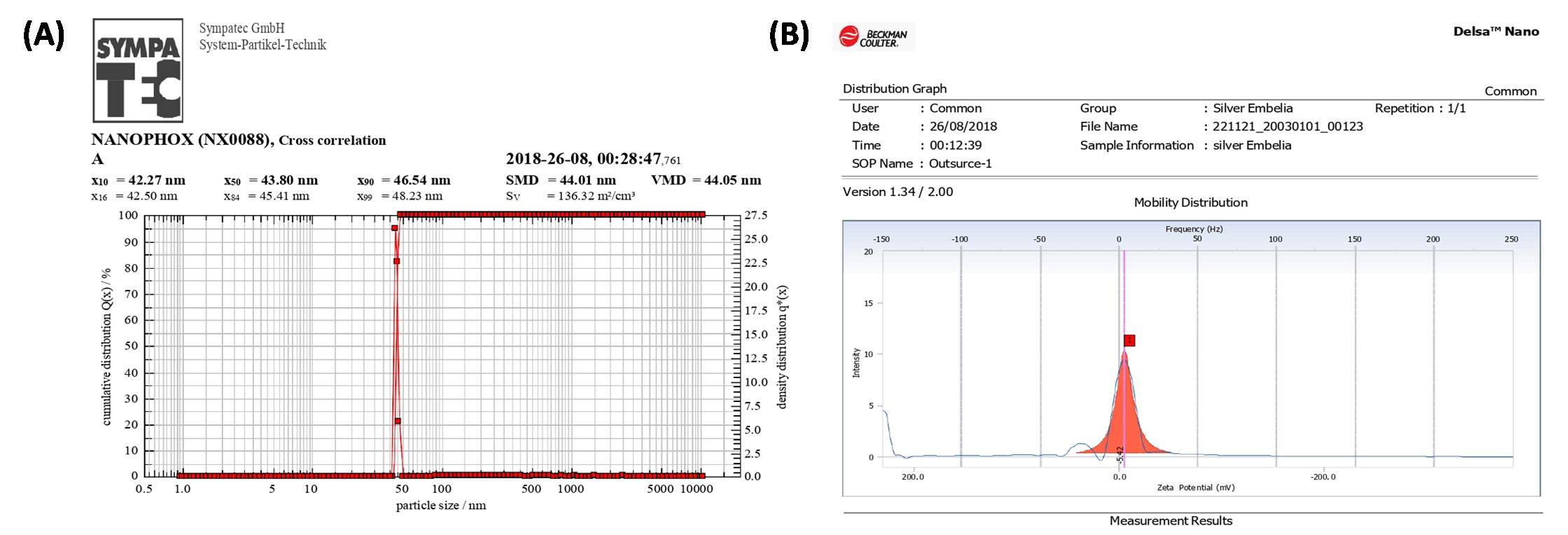

Supplement: Supplementary Figure 4 — (A) Cross-correlation for particle size analysis [NANOPHOX (NX0088)]; (B) zeta potential of embelin AgNPs. [file Image_4.JPEG]

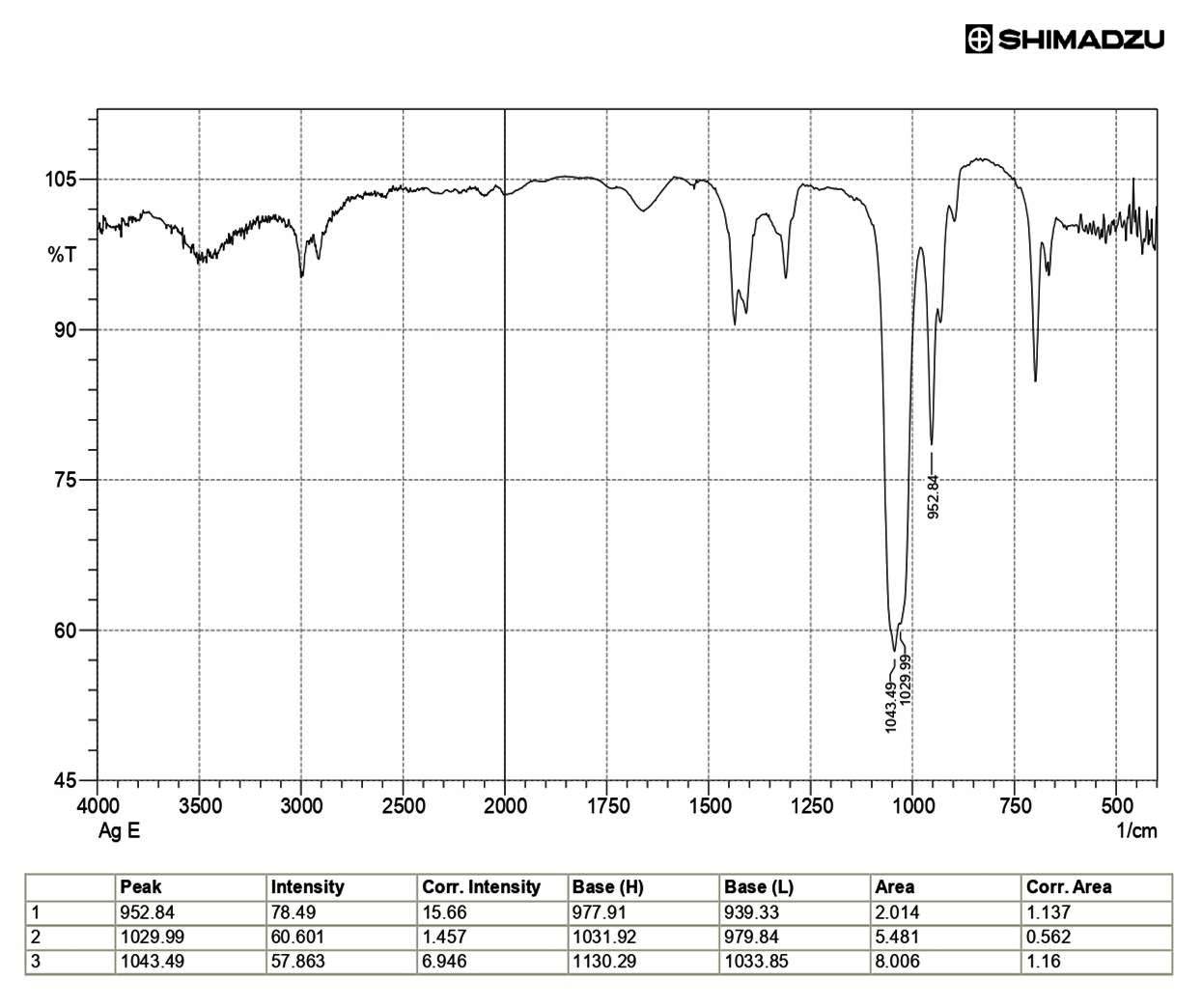

Supplement: Supplementary Figure 5 — FT-IR spectrum of embelin-derived AgNPs. [file Image_5.JPEG]
